# Assessment of spatial tumor heterogeneity using CT growth patterns estimated by tumor tracking on 3D CT volumetry of multiple pulmonary metastatic nodules

**DOI:** 10.1371/journal.pone.0220550

**Published:** 2019-08-01

**Authors:** Jeongin Yoo, Semin Chong, Changwon Lim, Miyoung Heo, In Gyu Hwang

**Affiliations:** 1 Department of Radiology, Seoul National University Hospital, Seoul, Korea; 2 Department of Radiology, Samsung Medical Center, Sungkyunkwan University School of Medicine, Seoul, Korea; 3 Department of Applied Statistics, Chung-Ang University, Seoul, Korea; 4 Division of Hematology/Oncology, Department of Internal Medicine, Chung-Ang University Hospital, Chung-Ang University College of Medicine, Seoul, Korea; INSERM, FRANCE

## Abstract

**Purpose:**

Our purpose was to assess the differences in growth rates of multiple pulmonary metastatic nodules using three-dimensional (3D) computed tomography (CT) volumetry and propose a concept of CT spatial tumor heterogeneity.

**Materials and methods:**

We manually measured the largest diameter of metastatic pulmonary nodules on chest CT scans, and calculated the 3D maximum diameter and the volume using a semi-automated 3D CT volumetry of each nodule. The tumor response was assessed according to the revised RECIST 1.1. We defined a nodule as an outlier based on 1.5 times growth during follow-up. The CT spatial tumor heterogeneity was statistically analyzed by the “minimum combination t-test method” devised in our study.

**Results:**

On manual measurement, the tumor response category was stable disease (SD) in all 10 patients. Of them, total 155 metastatic nodules (4–52 nodules per patient) were segmented using the 3D CT volumetry. In the 3D maximum diameter, 9 patients had SD except for one patient with partial response in the two selected nodules; for the volume, all 10 patients were SD. For the 3D maximum diameter, six patients had at least one outlier; whereas five patients had the outlier on the volume measurement. Six patients were proven to have overall CT spatial tumor heterogeneity.

**Conclusions:**

The spatial tumor heterogeneity determined in a CT parametric approach could be statistically assessed. In patients with CT spatial heterogeneity, tumors with different growth rates may be neglected when the nodules are assessed according to the current guideline.

## Introduction

In the RECIST 1.1 revised in 2009, five target lesions were selected instead of 10, and a maximum of two target lesions per organ were selected instead of five [1]. Measurable lesions were defined as those with a longest diameter of at least 10 mm on computed tomography (CT) with a section thickness of 5 mm or less, whereas non-measurable lesions were those with a longest diameter of less than 10 mm [2]. However, many viable tumor cells are still present in non-measurable lesions as well as non-target lesions. In addition, according to the concept of tumor heterogeneity, which implies the coexistence of subpopulations of cancer cells that differ in their genetic, phenotypic, or behavioral characteristics within a given primary tumor and between a given primary tumor and its metastatic lesions, only two randomly selected target lesions are not representative of the tumor burden [3]. This tumor heterogeneity may be present within a given tumor, such that different regions of the tumor harbor different repertoires of genetic aberrations (spatial heterogeneity), or during the course of disease progression (temporal heterogeneity) [3]. Hence, assessing the overall tumor burden by using only two lesions per organ has limited effectiveness, since pulmonary metastases frequently manifest as more than two nodules [4, 5].

Recently, precision medicine is an emerging field that focuses on identifying effective treatment approaches for patients based on genetic, environmental, and lifestyle factors [6, 7]. According to the concept of tumor heterogeneity, the gene mutation that occurs depending on the time and location of the tumor causes tumor recurrence, and decreases the antitumor therapeutic effect. Thus, the treatment and prevention based on precision medicine should include the assessment of tumor heterogeneity using noninvasive and ethical methods. This study attempted to review the possibility of considering spatial tumor heterogeneity in the course of treatment of patients with multiple lung metastases by acquiring the 3D maximum diameter and the volume of each lung nodule by using 3D CT volumetry.

In this study, we hypothesized that there would be a difference in the growth rates of all metastatic nodules because of spatial tumor heterogeneity when patients have multiple pulmonary metastatic nodules. Our purpose, therefore, was to measure the maximum diameter and volume of all multiple but countable metastatic pulmonary nodules by using a semi-automated three-dimensional (3D) volumetry software in each patient with pulmonary metastasis, assess the differences in growth rates of each metastatic nodule during follow-up, and propose a concept of CT spatial tumor heterogeneity.

## Materials and methods

The institutional review board of our institution approved this retrospective study and waived the requirement for informed patient consent for inclusion in this study.

A search of our institutional electronic medical records database yielded the data of 189 patients with multiple pulmonary metastases who had no significant change in tumor growth on follow-up chest CT after chemotherapy between January 2010 and December 2014. We retrospectively reviewed their clinical and chest CT findings. Of these 189 patients, those with less than 2 metastatic lung nodules or more than 60 (n = 92), or those with atelectasis due to pleural effusion (n = 81) or pneumothorax (n = 13) were excluded. Finally, 10 patients (M:F = 9:1; mean age, 66.9 years; range, 51–74 years) with multiple pulmonary metastases from thoracic or non-thoracic malignant tumors such as small cell lung cancer (n = 2), renal cell carcinoma (n = 3), maxillary sinus cancer (n = 1), colorectal adenocarcinoma (n = 3), and cholangiocarcinoma (n = 1) were enrolled in this study.

All CT examinations were performed at our institute by using a 64-slice or 256-slice multidetector CT system (Brilliance 64 or Brilliance iCT, respectively, Philips Healthcare). The scan parameters included a tube voltage of 120 kV and tube current of 120 mAs at a pitch of 1.015 for the 64-slice scanner and 0.915 for the 256-slice scanner. Single-phase peripheral intravenous power injection was performed using a total of 1.5 mL/kg of body weight of iopamidol-based nonionic contrast media (Pamiray 370, Dongkook Pharmaceutical). All CT data were reconstructed using a standard filter at a slice thickness of 1 mm.

### Manual measurement

A thoracic radiologist with 12 years of experience in thoracic imaging selected the two longest metastatic nodules of each patient and manually measured the diameters of the nodules on chest CT images by using an electronic caliper at the picture archiving and communication system (Maroview 5.4, Infinitt). In all 10 patients, the sums of the two longest diameters were used to assess the tumor response according to the response threshold based on the revised RECIST 1.1 [8]. Complete response (CR) was defined as the disappearance of all lesions. Progressive disease (PD) was defined as a more than 20% increase in the sum of the longest diameters of the target lesions, and partial response (PR) as a more than 30% decrease in the sum of the longest diameters of the target lesions. A patient who could not be classified as having either PR or PD was diagnosed as having stable disease (SD).

### 3D CT volumetry using a semi-automated method

**Fig 1:**
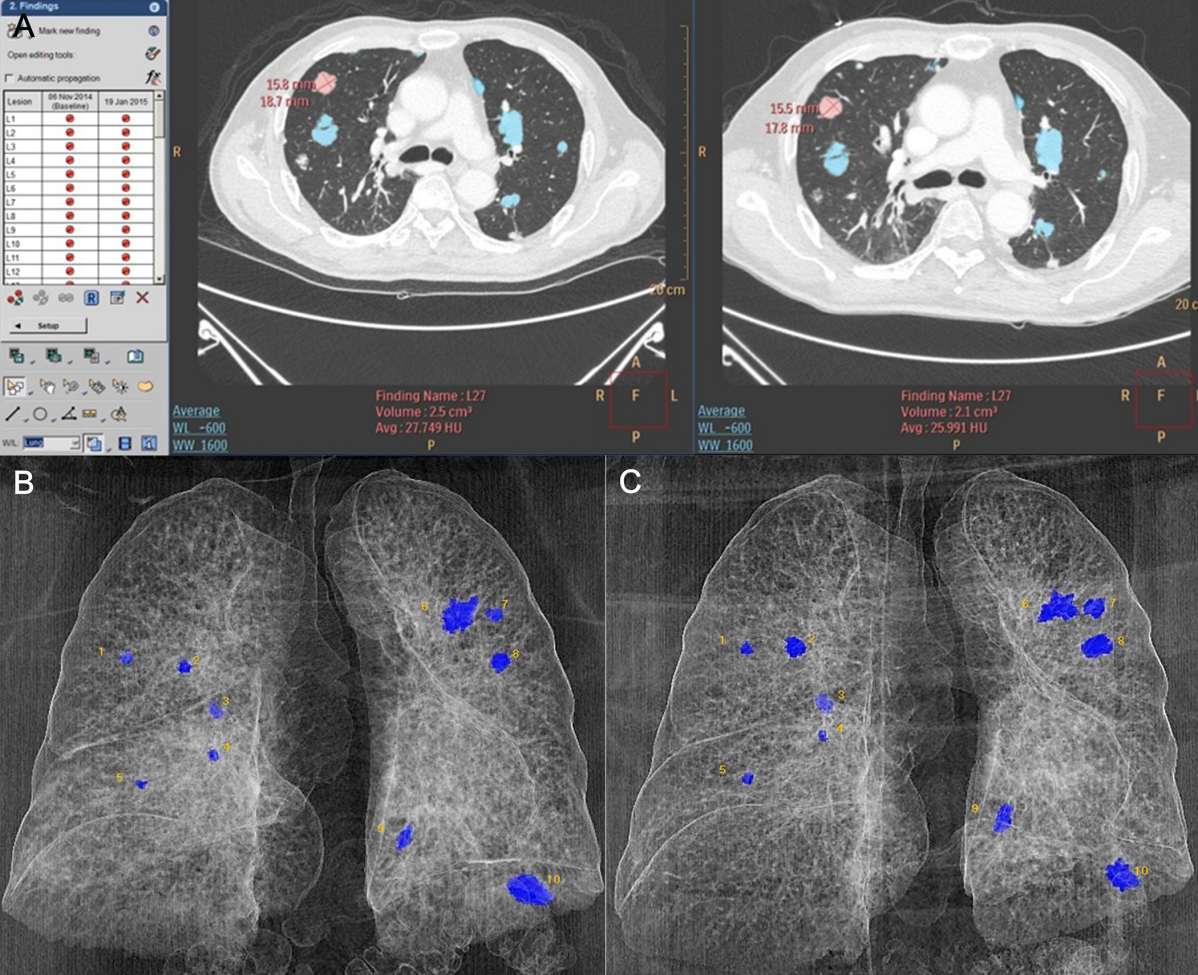
Lesion segmentation and tumor tracking by semi-automated 3D CT volumetry of multiple pulmonary metastatic nodules. (a) Metastatic pulmonary nodules are automatically segmented and manually edited on axial CT images of two time points using 3D visualization software. After segmentation, the 3D maximum diameter and volume of each nodule are automatically calculated. (b, c) Segmented pulmonary metastatic nodules are seen in 3D reconstructed images of baseline (b) and follow-up (c) chest CT scans.

All CT image data were transferred onto a dedicated workstation by using 3D visualization software (IntelliSpace Portal version 6.0, Philips Healthcare; EBW 4.5, Philips Healthcare) (Fig 1). Each patient’s metastatic nodules were automatically segmented using the “Segmentation” tool and manually edited on the axial CT images by a radiology resident. The segmentation and selective editing was finally confirmed by the experienced thoracic radiologist. After segmentation, the 3D maximum diameter (cm) and volume (cm^3^) of each nodule were automatically measured. The former was calculated as the longest diameter that can be drawn in the 3D volume, and the latter was calculated by counting the voxels in the contour. We calculated the number of segmented nodules and the time interval between the two serial CT examinations. We also calculated the change rates (%) of each nodule regarding both 3D maximum diameter and volume in each 10 patients and demonstrated them in bar graphs.

The two largest and total values of each parameter were selected for assessing the tumor response according to the revised RECIST 1.1 in all 10 patients. We calculated the change rate (%) in the sums of each parameter’s largest values between the two time points. For the 3D maximum diameter, the same response threshold as that for manual measurement was applied. For a volume change threshold, PD was defined as a more than 73% increase in the sum of the largest volume and PR as a more than 66% decrease in the sum of the largest volume [8].

We conducted a threshold analysis to determine a nodule that was 1.5 times larger on follow-up CT than on the previous CT, which was defined as an outlier. As change ratio criteria, 0.5 was adopted for 3D maximum diameters and 2.375 (calculated using the equation 1.5^3^–1^3^) for volumes in order to determine the outlier nodule presumably having prominent growth during the follow-up period.

### Statistical analysis

We compared the difference of change rates between the selected two largest and total nodules and between the 3D maximum diameter and volume using the paired t-test and the Bland-Altman plot. Statistical analyses were performed using MedCalc (version 12.6, MedCalc Software), PASW (version 18, SPSS), R (version 3.2.3) and Minitab (version 15, Minitab Inc.); p < 0.05 was considered statistically significant.

A statistical analysis for the concept of spatial tumor heterogeneity was performed by using the “minimum combination t-test method” devised by a statistician involved in our study. In this analysis, we considered the rate of change by using observations from two visits for the two variables calculated as follows:

$$rate\;of\;change=\frac{visit\;2-visit\;1}{visit\;1}\times 100\;(\%)$$

Under the assumption of no heterogeneity, we assumed that the average rates of change of each lesion were equal, and hence, the change rates of each lesion can be assumed to follow the same distribution. First, we divided the lesions of each patient into two groups of similar size (e.g., 31 lesions for a patient were divided into two groups of 15 and 16 lesions each). We considered all possible combinations of two groups, and not just certain combinations. For example, the number of combinations of dividing 31 lesions into 15 and 16 is _31∁15_ = 300,540,195 (for the convenience of analysis, the number of combinations was limited to a maximum of 4,000). Since the lesions were then divided into two groups for each combination, we could conduct a two-sample t-test for the rate of change. The hypotheses of the t-test were as follows:

$$H_{0}:\;\mu_{1}=\mu_{2}\;vs.\;H_{1}:\;\mu_{1}\neq \mu_{2}$$

where *μ*_1_ and *μ*_2_ were the average rates of change of groups 1 and 2, respectively. After conducting the two-sample t-test for a rate of change, if the p-value was less than the significance level, the null hypothesis was rejected and we concluded that the average rates for the two groups were different from each other. This finding suggested the existence of tumor heterogeneity. Since we should consider all possible combinations, we conducted the t-test as many times as the number of combinations, and hence, the same number of p-values was obtained. If the minimum value of the p-values was less than the significance level of 0.05, we rejected the overall null hypothesis of no heterogeneity. Thus, the existence of tumor heterogeneity regarding each variable for a patient could be observed. Thereafter, we conducted the t-test for the two rates of change and calculated two minimum p-values. Using those minimum p-values, we determined whether there existed any tumor heterogeneity regarding the corresponding variables for each patient. Lastly, if the minimum p-values were less than the significance level of 0.05, we made an overall conclusion that there existed spatial tumor heterogeneity in a patient.

## Results

The clinical and manual CT characteristics of all 10 patients are summarized in Table 1. In these 10 patients, the mean time interval between the two time points was 70.3 days (range, 32–151 days). According to the RECIST 1.1, the overall tumor response was assessed as SD by manual measurement in all 10 patients.

**Table 1:** The clinical features and tumor response assessment by manual CT measurement in 10 patients with multiple pulmonary metastases.

Patient No.	Sex	Age	Primary tumor	Clinical course	CT time interval (day)	Sum 1 (cm)	Sum 2 (cm)	Change^a^ (%)	Response assessment
1	F	66	Cholangiocarcinoma	Supportive care alone after PD for capecitabine and oxaliplatin 6^th^ cycle	88	2.2	2.2	0.0	SD
2	M	67	RCC	Sunitinib; 7^th^ cycle	32	1.4	1.4	0.0	SD
3	M	51	AD, rectal	Irinotecan and capecitabine; 3^rd^-4^th^ cycle	42	2.6	2.4	-7.7	SD
4	M	74	SCLC	Cisplatin and etoposide; 1^st^-2^nd^ cycle	53	4.1	2.9	-29.3	SD
5	M	56	AD, colon	Capecitabine; 7^th^-9^th^ cycle	69	2.9	2.5	-13.8	SD
6	M	71	SCLC	Paclitaxel; 3^rd^ -4^th^ cycle	52	6.3	6.2	-1.6	SD
7	M	69	RCC	Interleukin-2; 3^rd^ cycle	151	3.3	2.9	-12.1	SD
8	M	73	AD, colon	Capecitabine; 6^th^-8^th^ cycle	54	3.3	3.6	9.1	SD
9	M	74	RCC	Interferon; 2^nd^-4^th^cycle	88	5.5	5.8	5.5	SD
10	M	68	Maxillary sinus cancer	Cisplatin and 5-FU; 5^th^ cycle	74	6.2	5.9	-4.8	SD

CT = computed tomography RCC = renal cell carcinoma SCLC = small cell lung cancer AD = adenocarcinoma

Sum 1 = the sum of diameters of two target nodules at the first CT examination

Sum 2 = the sum of diameters of two target nodules at the follow-up CT examination

^a^ % change was calculated by the change rate of the sums of two largest values between the two time points based on RECIST 1.1. If the lesion grows, it is recorded as a positive number. If the lesion gets shrinkage, it is recorded as a negative number. Zero means neither growing nor shrinkage. SD = stable disease PD = Progression of disease

**Fig 2:**
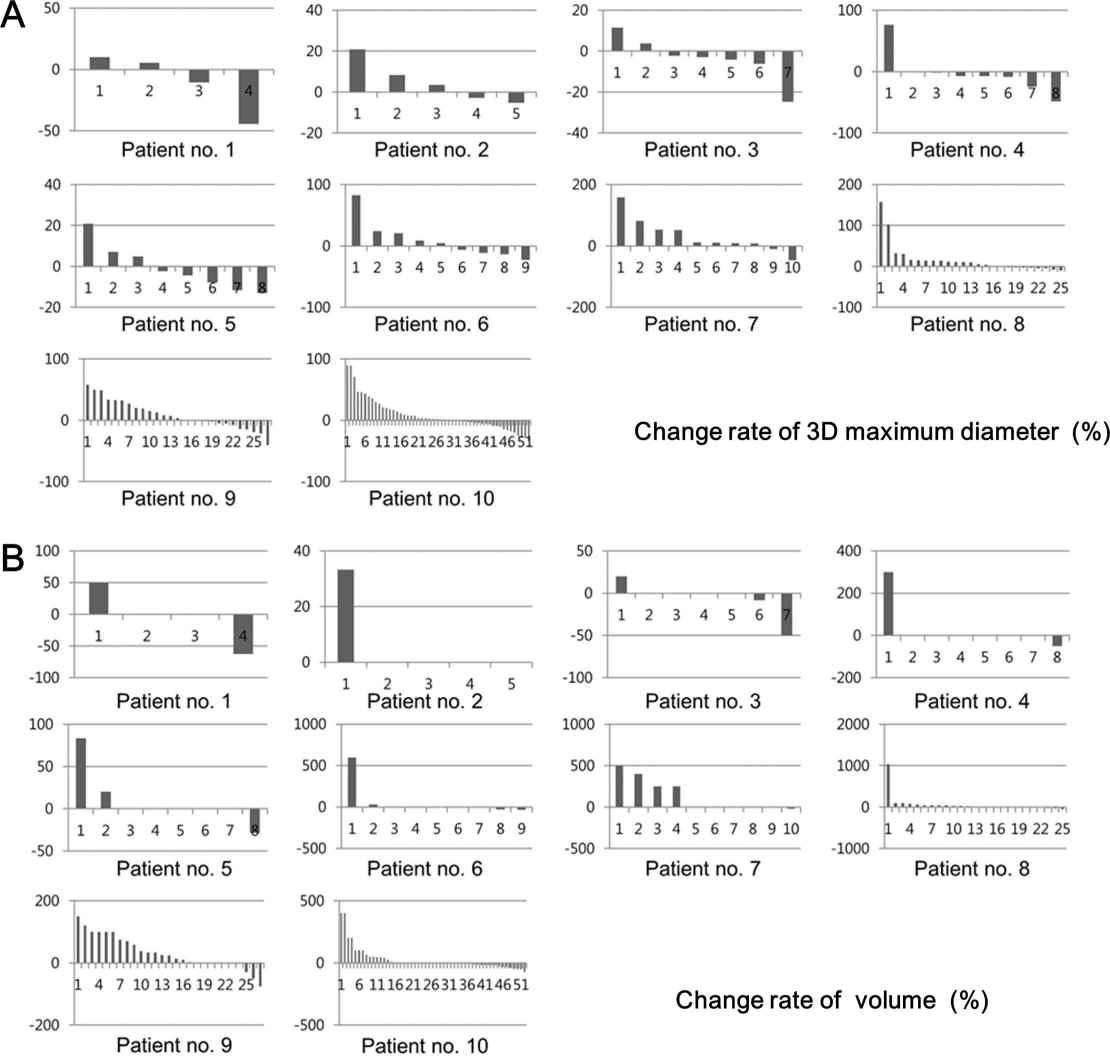
Change rates in 3D maximum diameter and volume of each nodule of 10 patients. Bar graphs show the change rates of each nodule in each patient regarding 3D maximum diameter (a) and volume (b). The x-axis of each patient’s graph means the number of nodules and the y-axis of each patient’s graph means the change rate (%) of each nodule.

In all 10 patients, total 155 metastatic pulmonary nodules (mean, 15.5 nodules; range, 4–52 nodules) were segmented and analyzed using semi-automated 3D CT volumetry. Regarding 3D maximum diameter and volume, the change rates (%) of each nodule in each patient were demonstrated in bar graphs (Fig 2). Table 2 summarizes the tumor response assessment according to the RECIST based on the number of metastatic nodules (two largest versus total nodules) measured by semi-automated 3D CT volumetry for the 3D maximum diameter and the volume. Regarding the 3D maximum diameter, nine patients were assessed as having SD in cases of both the two largest and all metastatic nodules, except for one patient (patient no. 4) who was assessed as having PR only in case of the two largest nodules but SD in case of all metastatic nodules. Regarding the volume, all 10 patients were assessed as having SD regardless of the number of selected nodules.

**Table 2:** Tumor response assessment based on the number of metastatic nodules measured by semi-automated 3D CT volumetry.

Patient No.	No. of metastatic nodules	No. of selected nodules	3D maximum diameter				Volume			
			Sum 1 (cm)	Sum 2 (cm)	Change^a^(%)	Response	Sum 1 (cm^3^)	Sum 2 (cm^3^)	Change^a^(%)	Response
1	4	Two	3.2	2.4	-25.0	SD	1.2	0.9	-25.0	SD
		Total	4.7	3.9	-17.0	SD	1.4	1.1	-21.4	SD
2	5	Two	3	2.9	-3.3	SD	1.3	1.3	0.0	SD
		Total	5.4	5.6	3.7	SD	1.8	1.9	5.6	SD
3	7	Two	3.6	3.7	2.8	SD	1.7	1.7	0.0	SD
		Total	7.3	7.2	-1.4	SD	2.3	2.2	-4.3	SD
4	8	Two	3.2	2.1	-34.4	PR	0.3	0.5	66.7	SD
		Total	7.7	6.9	-10.4	SD	0.9	1.1	22.2	SD
5	8	Two	3.3	2.9	-12.1	SD	1.3	1.6	23.1	SD
		Total	11.2	11.1	-0.9	SD	4.4	4.8	9.1	SD
6	9	Two	6.5	5.8	-10.8	SD	10.6	9.2	-13.2	SD
		Total	12.4	12.8	3.2	SD	11.9	11.7	-1.7	SD
7	10	Two	4.9	3.7	-24.5	SD	3.6	3.3	-8.3	SD
		Total	12.6	14.7	16.7	SD	5.0	7.1	42.0	SD
8	25	Two	4	4.3	7.5	SD	25.0	26.0	4.0	SD
		Total	27.7	31.9	15.2	SD	10.4	15.5	49.0	SD
9	27	Two	8.2	8.1	-1.2	SD	23.0	30.1	30.9	SD
		Total	45.7	48	5.0	SD	46.5	60.2	29.5	SD
10	52	Two	7	6.9	-1.4	SD	16.5	13.6	-17.6	SD
		Total	75.9	79.2	4.3	SD	62.0	59.9	-3.4	SD

Sum 1 = the sum of diameters or volumes of selected target nodules at the first CT examination

Sum 2 = the sum of diameters or volumes of selected target nodules at the follow-up CT examination

^a^ % change was calculated by the change rate of the sums of the selected largest values between the two time points based on RECIST 1.1. If the lesion grows, it is recorded as a positive number. If the lesion gets shrinkage, it is recorded as a negative number. Zero means neither growing nor shrinkage. SD = stable disease PR = partial response

**Fig 3:**
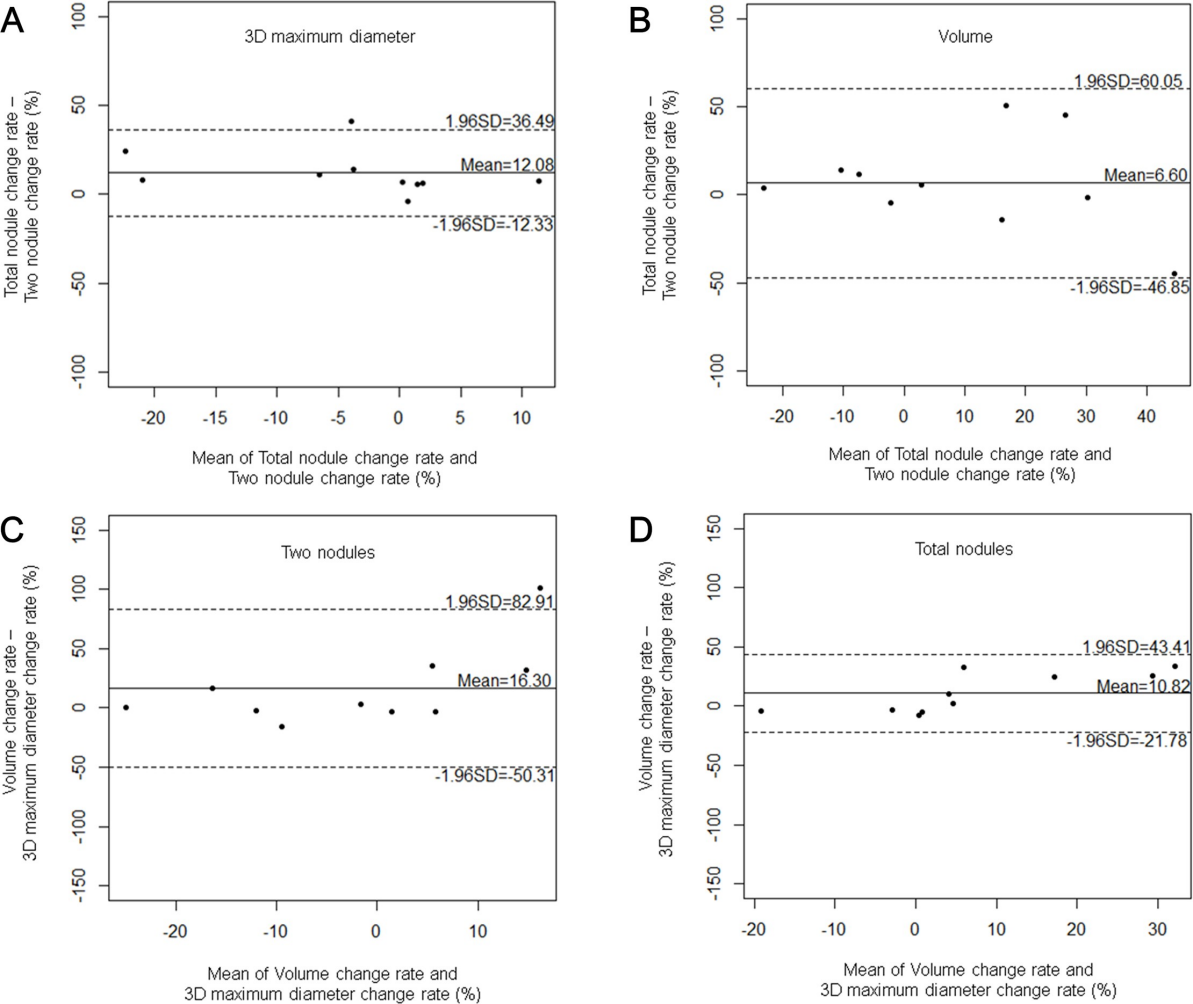
Bland-Altman plots of change rates between two time points according to the number of selected nodules and the measurement method. Plots show differences of change rates between two and total nodules estimated by 3D maximum diameter (a) and volume (b) measurements. Plots show differences of change rates between 3D maximum diameter and volume measurements in the selection of two (c) and total (d) nodules.

There was a statistically significant difference of the change rate between 3D maximum diameters of two and total nodules (p = 0.013); whereas there were no statistical significances in those between two and total nodule volumes (p = 0.464) and between the 3D maximum diameter and the volume of either two or total nodules (p = 0.164 and 0.070, respectively). On Bland-Altman plots, however, there were discrepancies between change rates of the measurement values based on the number of selected nodules and the measurement method (Fig 3). Regarding the mean difference of change rates between two and total nodules, the limit of agreement (i.e., mean ± 1.96 standard deviation) was wider on the volume measurement (-46.85 and 60.05) than that on the 3D maximum diameter measurement (-12.33 and 36.49) (Fig 3A and 3B). Regarding the mean difference of change rates between the measurement values estimated by the 3D maximum diameter and the volume, the limit of agreement was narrower in the selection of total nodules (-21.78 and 43.41) than that in the selection of two nodules (-50.31 and 82.91) (Fig 3C and 3D).

**Fig 4:**
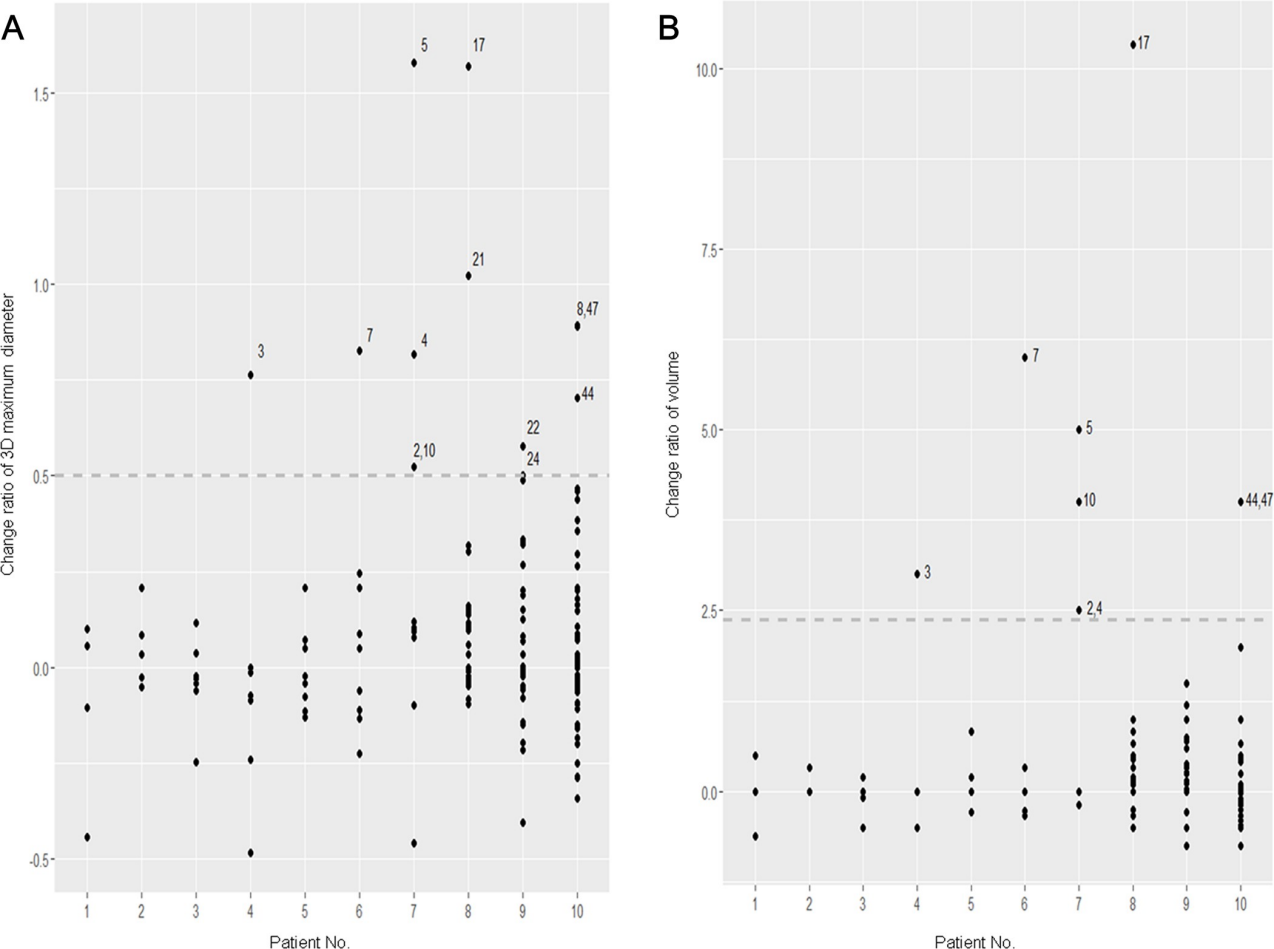
Change ratios between two time points estimated by linear and volumetric measurements. Scatter plots show change ratios of the 3D maximum diameter (a) and volume (b) of each nodule between two serial CT examinations. Dotted lines represent the change ratio criteria defined as 0.5 for 3D maximum diameter and 2.375 for volume in order to determine outlier nodules. Numbers above the dots are the nodule number given when performed segmentation.

The change ratio in each parameter between the two serial CT examinations is shown in Fig 4. For the 3D maximum diameter, six patients had at least one more up to four metastatic nodules with a change ratio of more than 0.5 (outliers); whereas the remaining four patients had metastatic nodules with a change ratio of less than 0.5 (Fig 4A). Regarding the volume, five patients had outliers with a change ratio of more than 2.375 (Fig 4B).

The CT spatial tumor heterogeneity of all metastatic nodules in each patient is shown in Table 3. Six patients (patients no. 5–10) were proven to have overall spatial tumor heterogeneity by using the minimum of the two minimum p-values less than 0.05, while the other four patients (patients no. 1–4) had no overall spatial tumor heterogeneity. A minimum p-value less than 0.05 was obtained in 6 patients (patients no. 5–10) for the 3D maximum diameter and in three patients (patients no. 7, 9, and 10) for the volume, which was presumed to have spatial tumor heterogeneity based on the measurement method.

**Table 3:** CT spatial tumor heterogeneity of each patent’s all metastatic nodules in 10 patients.

Patient No.	No. of nodules	3D maximum diameter (cm)	Volume (cm^3^)	Min(min(p))	Overall spatial heterogeneity
1	4	0.2815	0.3005	0.2815	No
2	5	0.0966	0.4226	0.0966	No
3	7	0.0879	0.1879	0.0879	No
4	8	0.1463	0.3292	0.1463	No
5	8	0.0324	0.1955	0.0324	Yes
6	9	0.0399	0.2978	0.0399	Yes
7	10	0.0305	0.0283	0.0283	Yes
8	25	0.0273	0.1052	0.0273	Yes
9	27	0.0003	0.0002	0.0002	Yes
10	52	0.0003	0.0033	0.0003	Yes

Note—Min(min(p)) = minimum of minimum p-value among two variables.

## Discussion

Few studies have investigated the optimal number of target lesions required for the objective assessment of tumor response. Marten et al. suggested that the assessment of tumor response using volume criteria in pulmonary metastases should include a minimum of three target lesions, on the basis of their analysis of five metastatic lesions [9]. In our study, we conducted linear and volumetric quantification by using semi-automated 3D CT volumetry of more than four metastatic lung nodules, which, to our knowledge, is the first study on tumor response assessment by measuring all of the metastatic nodules (up to 52 nodules) in each patient. For linear quantification of metastatic nodules based on the 3D maximum diameter estimated by semi-automated 3D CT volumetry, there was discrepancy in overall response between the two largest and all metastatic nodules in one patient (patient no. 4), whereas all 10 patients showed SD for volume quantification regardless of the number of nodules (Table 2). This discrepancy may affect the oncologist’s decision to continue the current chemotherapeutic agent or to replace it with another drug. Therefore, we think that determining whether the tumor response to the two largest lesions or all of the lesions actually reflects a patient’s current disease status is an important issue related to future treatment strategies and patient prognosis.

Recently, tumor heterogeneity is perceived as one of the causes of resistance to targeted therapy, which can be generally evaluated by a genetic approach [10]. However, there are practical limitations to the genetic evaluation of all metastatic nodules in each patient. In our study, for the practical characterization of spatial heterogeneity, we devised a CT phenotypic approach, which implied the measurement of morphologic changes of a nodule observed on CT as phenotypes resulting from the expression of genes in a nodule. To analyze the presence or absence of this CT spatial tumor heterogeneity statistically, we also devised the so-called minimum combination t-test method. In our study, six patients were statistically proven to have overall CT spatial tumor heterogeneity of metastatic nodules. Therefore, in patients with spatial tumor heterogeneity assessed by the CT phenotypic approach, when only two target lesions will be assessed according to the RECIST 1.1, it is not likely to represent a change in overall tumor burden of metastatic nodules during chemotherapy. Hence, such cases might be assessed by measuring all metastatic nodules based on the volume calculated using 3D CT volumetry. This is a major clinical implication of our study, which recommends a change to the current methodology of assessment of tumor response using the linear measurement of a few target lesions.

In terms of primary tumors of the lung cancer, tumor heterogeneity has been assessed noninvasively using variable imaging modalities and features. The texture analysis refers to a variety of mathematical methods that can be used to evaluate the gray-level intensity and position of the pixels within an image to derive texture features that provide a measure of intralesional heterogeneity [11]. However, these texture analyses would not be applicable in cases of multiple pulmonary metastatic nodules due to the lesion size and number. In our study, we found out nodules that grow more than 1.5 times in 3D maximum diameters and volumes and described them as outliers (Fig 4). We think that these outliers could have spatial tumor heterogeneity which results in different growth rates and patterns among multiple metastatic nodules.

Nevertheless, our study has several limitations. First, our study included a small number of patients. However, as our study focused on the growth rate of each lung nodule, the total number of nodules would be more meaningful than the number of patients. We have applied a minimal method to establish a hypothesis that metastatic nodules are heterogeneous within each patient rather than heterogeneous in each patient, resulting in spatial tumor heterogeneity. Second, our study population had diverse primary tumor entities and subsequently underwent treatment using variable chemotherapeutic regimens. We think that the type of primary tumor would have little effect on the results because our study was about the tumor response assessment in patients with multiple pulmonary metastases, which have been reported to show no interval change. Third, each nodule in our patients was not pathologically proven metastatic and was not genetically confirmed to have spatial tumor heterogeneity. However, it is not practically and ethically possible to perform a biopsy for all metastatic nodules at present. Fourth, some of the follow-up intervals were relatively short. However, this is unlikely to affect the evaluation of tumor response determined using two different measurement techniques.

## Conclusions

The volume calculated using 3D CT volumetry might be more reliable than that calculated using the traditional methods for tumor response assessment. In our study, spatial tumor heterogeneity determined via the CT phenotypic approach could be statistically assessed using the minimum combination t-test method. In patients with CT spatial heterogeneity, the outlier tumor with a different growth pattern may be excluded when only two or some target lesions are assessed according to the current guideline. Therefore, we expect that these outlier tumors would be the emerging targets that necessitate a different treatment strategy in the future.
